# Moving towards adaptive management of cyanotoxin‐impaired water bodies

**DOI:** 10.1111/1751-7915.12383

**Published:** 2016-07-15

**Authors:** Hans W. Paerl, Timothy G. Otten, Alan R. Joyner

**Affiliations:** ^1^ Institute of Marine SciencesUniversity of North Carolina at Chapel HillMorehead CityNCUSA; ^2^ Bend Genetics, LLC87 Scripps Drive, Ste. 301SacramentoCAUSA

## Abstract

The cyanobacteria are a phylum of bacteria that have played a key role in shaping the Earth's biosphere due to their pioneering ability to perform oxygenic photosynthesis. Throughout their history, cyanobacteria have experienced major biogeochemical changes accompanying Earth's geochemical evolution over the past 2.5+ billion years, including periods of extreme climatic change, hydrologic, nutrient and radiation stress. Today, they remain remarkably successful, exploiting human nutrient over‐enrichment as nuisance “blooms.” Cyanobacteria produce an array of unique metabolites, the functions and biotic ramifications of which are the subject of diverse ecophysiological studies. These metabolites are relevant from organismal and ecosystem function perspectives because some can be toxic and fatal to diverse biota, including zooplankton and fish consumers of algal biomass, and high‐level consumers of aquatic food sources and drinking water, including humans. Given the long history of environmental extremes and selection pressures that cyanobacteria have experienced, it is likely that that these toxins serve ecophysiological functions aimed at optimizing growth and fitness during periods of environmental stress. Here, we explore the molecular and ecophysiological mechanisms underlying cyanotoxin production, with emphasis on key environmental conditions potentially controlling toxin production. Based on this information, we offer potential management strategies for reducing cyanotoxin potentials in natural waters; for cyanotoxins with no clear drivers yet elucidated, we highlight the data gaps and research questions that are still lacking. We focus on the four major classes of toxins (anatoxins, cylindrospermopsins, microcystins and saxitoxins) that have thus far been identified as relevant from environmental health perspectives, but caution there may be other harmful metabolites waiting to be elucidated.

## Introduction

Cyanobacteria are the Earth's oldest known prokaryotic oxygenic phototrophs, with fossil evidence pointing to their presence in the Proterozooic, some 2.5 billion years ago (Schopf, 2000). This period witnessed the transition from anoxic to oxic conditions, in large part due to their photosynthetic activities. They have also experienced periods of varying nutrient (N, P, C and minor elements) abundance and availability, and a great deal of variability in climatic conditions, including extremely wet and dry periods, combined with major changes in the Earth's surface temperature. Lastly, major geophysical events such as volcanism and continental drift have altered their habitats and have exerted ecophysiological constraints over a wide range of time scales. As such, cyanobacteria have experienced the full spectrum of physical–chemical–biotic changes that have impacted the Earth's biosphere; unsurprisingly, cyanobacteria exhibit an extremely broad geographic distribution, ranging from polar to tropical regions, and from subsurface aquatic to alpine habitats (Potts and Whitton, 2000). Across this broad range, they inhabit virtually all terrestrial and aquatic habitats, ranging from deserts to tropical rain forests and from the ultraoligotrophic open ocean to hypereutrophic lakes (Potts and Whitton, 2000; Whitton, 2012). Lastly, cyanobacteria exhibit a remarkable ability to both counter extreme climatic conditions and to thrive under them.

Within these diverse habitats, cyanobacteria possess widespread adaptations to climatic extremes, including the formation of heat and desiccation‐tolerant resting cells, or akinetes, the presence of photo‐protective pigments and desiccation‐resistant sheaths and capsules, the ability to glide on surfaces to adjust their position within a bloom and in the water column (by buoyancy regulation) in response to irradiance and nutrient gradients (Potts and Whitton, 2000; Reynolds, 2006). They have also developed a wide array of physiological adaptations to cope with periods of nutrient limitation, including the ability to sequester (by chelation) iron (Wilhelm and Trick, 1994), efficiently fix gaseous (CO_2_) and dissolved inorganic carbon (DIC; Paerl and Millie, 1996), store phosphorus, nitrogen (N_2_) and other essential nutrients (Healy, 1982; Reynolds, 2006), and for some (the Nostocales, some Oscillatoriales and some picocyanobacterial genera) the ability to convert or “fix” atmospheric N_2_ into biologically available ammonia (Gallon, 1992). Many cyanobacterial genera have formed mutualistic and symbiotic associations with fungi, algae, higher plants and animals, enabling them and their partners to exploit and thrive in potentially hostile and extreme environments (Paerl, 1982; Raven, 2002).

The diverse and remarkable physical–chemical and biotic adaptations that cyanobacteria utilize make for an extensive repertoire of ecological strategies aimed at surviving and at times thriving, as nuisance blooms, under a wide range of environmental conditions, including human alterations of aquatic environments; e.g. nutrient over‐enrichment (eutrophication), hydrologic alterations due to water withdrawal (for drinking, irrigation, industrial use) from streams, rivers and lakes, dam/reservoir, artificial waterway construction, and perturbations of benthic and planktonic habitats.

Lastly, and central to this article, cyanobacteria produce a wealth of secondary metabolites of which the functions and biotic ramifications remain largely unknown. Some of these metabolites have come to light in an environmental and societal context because they can have harmful effects on organismal and ecosystem function, as toxic substances that adversely affect diverse biota, including zooplankton and herbivorous fish, bioaccumulate in higher trophic levels and directly impair drinking water. It is hypothesized that the so‐called “cyanotoxins” serve ecophysiological functions aimed at optimizing growth and protecting cyanobacterial cells during periods of environmental stress and constraints as outlined above (e.g. Paerl and Millie, 1996), rather than specifically serving as defensive (i.e. anti‐grazing) compounds directed at high‐level consumers. This is based on numerous field and laboratory observations that indicate that higher ranked consumers are not necessarily a threat to cyanobacterial blooms nor are they able to effectively control the global proliferation that we are currently experiencing (Paerl and Millie, 1996).

In this contribution, we will explore the molecular mechanisms underlying secondary metabolite and toxin production, environmental and cellular conditions controlling toxin production and, ecophysiological rationales for toxin production in aquatic ecosystems supporting their growth and proliferation. By linking molecular‐level mechanisms to their potential controls on microbial community and ecosystem scales, it is hoped that both the ecological reasons and potential environmental controls of toxin production can be uncovered and potentially utilized in aquatic ecosystem management schemes aimed at improving water quality and environmental health of impacted waters.

## Overview of cyanotoxins and their putative environmental drivers

### Anatoxins

Anatoxin‐a was identified over 40 years ago following a series of livestock poisoning events that were traced back to blooms of *Anabaena flos‐aquae* (this genus has recently been renamed *Dolichospermum*) (Devlin *et al*., 1977). Since then it has been identified in several families of cyanobacteria isolated from both pelagic and benthic environments. The neurotoxin is a potent nicotinic acetycholine receptor agonist that blocks neuromuscular junctions leading to rapid respiratory arrest. In addition to anatoxin‐a and its methylene homologue homoanatoxin‐a, additional variants include dihydroanatoxin‐a and dihydrohomoanatoxin‐a and their respective cis/trans isomers. All analogues are produced from a 10‐gene operon that includes three polyketide synthases (PKS), a type II thioesterase, a transporter, an acyl carrier and four tailoring enzymes (Méjean *et al*., 2009). The gene cluster organization and orientation varies by cyanobacterial genus, as evinced from a number of operons sequenced from *Anabaena*,* Oscillatoria*,* Cylindrospermum* and *Cuspidothrix* isolates (Méjean *et al*., 2014). Individual strains may produce more than one anatoxin‐a analogue simultaneously (Mann *et al*., 2012). Dihydroanatoxin‐a and dihydrohomoanatoxin‐a exhibit approximately 10‐fold lower binding affinities and toxicities than anatoxin‐a and homoanatoxin‐a based on mouse LD_50_ (Wood *et al*., 2012).

Studies investigating the environmental factors that influence regulation of this operon are generally lacking, but suggest that stimuli may be strain specific. Using cultures of planktonic *Anabaena* and *Aphanizomenon*, anatoxin‐a was shown to decrease in cells grown under high temperatures (up to 30°C), low (2 μmol m^−2^ s^−1^) or high (128 μmol m^−2^ s^−1^) light intensities, and replete N conditions, while orthophosphate concentration had no effect on toxin levels (Rapala *et al*., 1993). An investigation of N concentration on anatoxin‐a production in *Aphanizomenon issatschenkoi* found that moderate N stress coincided with maximal toxin production (Gagnon and Pick, 2012). A separate study of *Anabaena* sp. reported that anatoxin‐a was maximally produced under temperatures and light levels slightly suboptimal for growth (Rapala and Sivonen, 1998).

In a study of benthic *Phormidium autumnale* isolates, over 85% of total anatoxin was observed to occur intracellularly across a range of low to high N and P treatments (Heath *et al*., 2014). In that study, the authors also reported that dihydroanatoxin‐a concentrations decreased when N and P concentrations were elevated (21 mg l^−1^ and 3 mg l^−1^ respectively), whereas homoanatoxin‐a quota increased when P concentrations were reduced below 0.08 mg l^−1^. Potentially complicating the identification of environmental drivers was the observation of transcripts from several *ana* genes but an absence of detectable toxin in *Cuspidothrix* (formerly *Aphanizomenon*) *issatschenkoi* CHABD3 (Jiang *et al*., 2015); this raised the possibility that anatoxin‐a synthesis may be subject to post‐transcriptional regulation.

### Cylindrospermopsins

Cylindrospermopsin (CYN) is a guanidine alkaloid that exhibits broad spectrum cytotoxicity, as well as potent liver and kidney toxicity due to glutathione and protein synthesis inhibition, and the production of damaging cytochrome p‐450‐generated metabolites (Humpage *et al*., 2005). In addition to CYN, four other analogues have been identified; 7‐epi‐CYN is a C‐7 epimer of CYN with similar toxicity (Norris *et al*., 1999), 7‐deoxy‐CYN lacks the hydroxyl group on C‐7 and exhibits little to no toxicity in mice (Banker *et al*., 2001), and 7‐deoxy‐desulfocylindrospermopsin and 7‐deoxy‐desulfo‐12‐acetylcylindrospermopsin were recently identified and their relative toxicities remains undetermined (Wimmer *et al*., 2014). The *cyr* gene cluster is responsible for CYN biosynthesis and has been reported to consist of 11 genes in *Aphanizomenon* (Stüken and Jakobsen, 2010), *Oscillatoria* (Mazmouz *et al*., 2010) and *Raphidiopsis* (Jiang *et al*., 2012), but 15 genes in *Cylindrospermopsis* (Mihali *et al*., 2008), with significant rearrangements in gene order between genera, suggestive of substantial divergence from a common ancestor (Mazmouz *et al*., 2010), recombination and/or horizontal gene transfer (Cirés and Ballot, 2016). The operon encodes for non‐ribosomal peptide synthetase/PKS genes, transferases, uracil ring formation, an exporter and other tailoring functions. The conversion of 7‐deoxy‐CYN into CYN is controlled *in vitro* by a 2‐oxoglutarate‐dependent iron oxygenase encoded by *cyrI* (Mazmouz *et al*., 2010). This was corroborated in a strain of *Raphidiopsis curvata* (CHAB1150) that was found to contain an insertion mutation in *cyrI* that induced a frameshift and stop codons which resulted in a truncated product. As a result the strain could only produce 7‐deoxy‐CYN (Jiang *et al*., 2012).

Although the operon contains an exporter, most studies of *Cylindrospermopsis* suggest that the majority of total CYN is retained intracellularly (Saker and Eaglesham, 1999; Willis *et al*., 2015). On the contrary, investigations of CYN‐producing *Aphanizomenon* blooms in German lakes have reported that the majority of toxin occurred extracellularly (Rücker *et al*., 2007). However, as with any intracellular compound, cell senescence or lysis will result in larger percentages of extracellular toxins, additionally, the co‐occurrence of toxic and non‐toxic genotypes in natural settings may also complicate estimate of toxin quota. For example, other culture‐based studies have reported extracellular CYN fractions to range from 11% to 26% for *Aphanizomenon* (Preußel *et al*., 2006, 2009, 2014) and 52–62% for *Oscillatoria* (Mazmouz *et al*., 2010). Culture studies of *C. raciborskii* indicate that the highest extracellular CYN concentrations correspond with cells in stationary growth phase or during the end of a bloom (Dyble *et al*., 2006).

A clear understanding of environmental conditions that promote CYN production or toxic strain dominance is presently lacking. Two recent studies have reported that *cyr* gene expression and CYN production in *C. raciborskii* cultures do not vary in response to different nitrogen and phosphorus regimes, although one of the strains was used in both studies (Stucken *et al*., 2014; Willis *et al*., 2015). The observation that CYN was constitutively produced runs contrary to other studies that have reported differential CYN production and cell quotas in *Oscillatoria* (Bormans *et al*., 2013), *Aphanizomenon* (Preußel *et al*., 2014) and even other *Cylindrospermopsis* isolates (Dyble *et al*., 2006). Combined, these findings suggest that there may be species or strain‐level differences in CYN production possibly influenced by the physiological or environmental importance/function that CYN and its derivatives provide individual ecotypes.

The presence of two transcription start points between *cyrA* and *cyrC* (called *aoaA* and *aoaC* at the time) from *Aphanizomenon ovalisporum* was shown to be differentially expressed under N and light stress conditions (Shalev‐Malul *et al*., 2008). The authors hypothesized that there may be two promoters regulating transcription, one that functions constitutively and another that regulates transcription in response to specific environmental conditions. The authors also identified an AbrB‐like protein that binds between the transcription start points, and sequences for similar AbrB‐like proteins were subsequently identified from CYN‐producing strains of *Oscillatoria* and *Cylindrospermopsis* (Mazmouz *et al*., 2010) suggesting it may play a central role in *cyr* regulation. Congruent with the hypothesis that N and light limitation regulate CYN production, *Cylindrospermopsis* cultures grown in the absence of N have been shown to produce more CYN on a per cell basis (Saker and Neilan, 2001) and high light intensity has been found to reduce CYN production in both *Aphanizomenon* and *Cylindrospermopsis* (Dyble *et al*., 2006).

Regarding other environmental parameters, cultures of *Aphanizomenon ovalisporum* were shown to significantly decrease CYN content in response to sulfate and phosphate limitation (Bácsi *et al*., 2006). Semi‐continuous culture studies using two *Aphanizomenon* isolates grown under various light intensity (10–60 μE m^−2^ s^−1^) and temperature (16–25°C) regimes indicated that total CYN content exhibited little response relative to the light conditions tested (Preußel *et al*., 2009), although the light intensity may not have been high enough to repress *cyr* transcription. Regarding temperature, there was a 2.6‐fold reduction in total CYN at 25°C relative to the 16°C treatment for one of the strains tested, but not the other (Preußel *et al*., 2009). We note that higher temperatures have also been linked with lower CYN production in *C. raciborskii*, with complete abolishment of synthesis occurring at 35°C (Saker and Griffith, 2000).

Combined, these results indicate that there remains a need for further studies before the physiological basis for CYN production is unravelled, although studies to date suggest that CYN may have a role in response to N and light limitation. The finding that high temperatures abolish CYN production should also be further investigated, with particular attention given to the effect that temperature may have on other cellular factors that could indirectly influence CYN biosynthesis.

### Microcystins

The hepatotoxin microcystin (MC) is the best characterized cyanotoxin and also likely the most widely occurring throughout the phylum of cyanobacteria. MCs are synthesized via a bi‐directionally transcribed gene cluster (10 genes in *Microcystis* and *Anabaena*, 9 genes in *Planktothrix*) that spans ~55 kb and consists of multiple non‐ribosomal peptide synthetase and PKS genes, an ABC transporter and other genes involved in other tailoring functions (Tillett *et al*., 2000; Christiansen *et al*., 2003; Rouhiainen *et al*., 2004). Although the *mcy* operon contains a ABC transporter, there is no evidence of active export of MC from the cells (Rohrlack and Hyenstrand, 2007). The *mcy* genes in each genus have unique arrangements, suggestive of a history of homologous recombination and evolutionary decent from a common MC‐producing ancestor (Rantala *et al*., 2004); however, there has yet to be any evidence indicating that *mcy* genes are horizontally transferred across genera.

There are over 100 congeners of MC that have been identified to date (Puddick *et al*., 2013; Qi *et al*., 2015). All congeners consist of a general cyclic heptapeptide structure (cyclo‐d‐Ala‐**X**‐d‐MeAsp‐**Z**‐Adda‐d‐Glu‐Mdha) with two positions (X and Z) subject to variable L‐amino acid incorporation, for which there are at least 15 and 12 amino acid variations, respectively, that have been reported (Hoeger *et al*., 2007). In addition to amino acid substitution, structural modifications via methylation/demethylation and esterification increase the number of possible congeners that can be produced. Each congener exhibits different toxicities in mammals, with MC‐LR variants considered to be among the most toxic and commonly encountered (Shimizu *et al*., 2014). Once ingested, MCs are taken up by the bile acid transport system where they bind selectively to protein phosphatase 1 and 2A in hepatocytes, resulting in severe damage to the liver (Runnegar *et al*., 1993); chronic exposure to MCs has also been linked to tumours and hepatocarcinoma (Nishiwaki‐Matsushima *et al*., 1992; Falconer and Humpage, 1996).

Studies using batch cultures of cyanobacteria have shown that MC production is highest when nitrogen and phosphorus concentrations are highest (Sivonen, 1990; Vezie *et al*., 2002; Harke and Gobler, 2013). However, others have argued that cellular growth rate may be the primary factor influencing MC production, with maximal toxin production typically coinciding during periods of maximal growth rate (Orr and Jones, 1998; Oh *et al*., 2000). As growth rates are highest when nutrient conditions are non‐limiting–assuming adequate light, temperature and micronutrients, these findings seem to be corroborative. Harke and Gobler (2013) used metatranscriptomics to assess how a cultured isolate of *M. aeruginosa* (LE‐3) responds to N or P stress and observed a significant decrease in MC and *mcy* transcription under low dissolved inorganic N. However, on a per cell basis, MC production levels have also been shown to increase during suboptimal growth periods, such as when cells are stressed due to low iron availability (Utkilen and Gjolme, 1995) or too high light intensity (Kaebernick and Neilan, 2001). Complicating matters, Fe uptake is highest under high light due to its key role in photosynthesis (Lukac and Aegerter, 1993); therefore, iron homoeostasis and cellular redox state may ultimately control MC production (Kaebernick and Neilan, 2001). Using comparative proteomics, Alexova *et al*. (2011a) showed that *mcy*+ strains of *Microcystis* differentially express proteins involved in carbon metabolism and redox balance and that *mcy* transcription is highest during periods of Fe‐limitation (Alexova *et al*., 2011b).

Other studies have investigated the role of oxidative stress on MC function by comparing a MC‐producing strain of *M. aeruginosa* (PCC7806) with *mcy* knockout variants of the strain. One such study compared the growth rate of the wild‐type (WT) toxic strain with the *mcy*‐deficient mutant (MT) across a range of low to high light (16–700 μmol photons m^−2^ s^−1^) conditions and from low to high concentrations of hydrogen peroxide (H_2_O_2_; 0–1 μmol) in order to induce oxidative stress in the cultures (Zilliges *et al*., 2011). The authors observed pigment chlorosis in the MT strain under both high light and high H_2_O_2_ conditions, whereas the WT strain exhibited no negative effects; this suggested that MCs may serve an intracellular protective role during periods of photooxidative stress. Indeed, UV‐B radiation has been shown to more negatively affect non‐toxic strains of *Microcystis* than MC‐producing ones (Yang *et al*., 2015). Further studies using immunolabeling techniques have shown that MCs bind to and protect carboxysome and phycobilisome proteins from reactive oxygen species (ROS) during periods of photooxidative stress (Zilliges *et al*., 2011). This typically occurs in early bloom stages when photosynthetic growth rates and biomass accumulation are highest, and oxygen supersaturation and production of ROS (e.g. H_2_O_2_, O_2_^−^) is greatest.

In addition to their role in redox balance, there appear to be other functions for MCs. A study assessing competitive dominance of toxic and non‐toxic strains of *M. aeruginosa* used a *mcy*‐producing wild‐type (WT) strain and its *mcy*‐deficient mutant to assess competition for CO_2_, and found that the WT strain outcompeted the non‐toxic strain under low CO_2_ concentrations, but not high CO_2_ concentrations (Van de Waal *et al*., 2011). These results were similar to another competition study that also found the non‐toxic variant able to outcompete the toxic wild‐type strain under higher CO_2_ conditions (Jahnichen *et al*., 2007). Higher temperatures have been shown to promote the dominance of MC‐producing strains of *Microcystis* over their non‐toxic counterparts (Davis *et al*., 2009). Considering that CO_2_ more readily dissolves into solution at lower temperatures, the dominance of toxigenic strains under low CO_2_/DIC conditions may be interconnected with temperature.

Interestingly, studies indicate that N availability may influence MC production differently between N_2_‐fixing and non‐fixing genera; the diazotrophs *Anabaena*,* Aphanizomenon* and *Nodularia* exhibit higher toxin production rates in N‐free media, whereas *Oscillatoria* and *Microcystis* have higher rates under N replete conditions (Kaebernick and Neilan, 2001). In contrast, growth competition studies using both *Planktothrix* and *Microcystis* found that MC‐producing strains tend to outcompete non‐toxic strains under low N conditions (Briand *et al*., 2008). However, these genera diverge in that toxigenic *Microcystis* tends to outcompete non‐toxic strains under high light conditions when the opposite has been shown for *Planktothrix agardhii* (Briand *et al*., 2008). Compared with *Microcystis* and *Planktothrix* (Oscillatoriales), studies investigating MC production by N_2_‐fixing genera are generally lacking, and especially so with regard to the effects of iron or photooxidative stress on toxin production. The finding that MC production rates are higher during periods of N‐stress in diazotrophic cyanobacteria indicates that there may be other drivers of toxicity that require further study for this group. Lastly, even though most MCs are retained intracellularly, the presence of an ABC transporter within the *mcy* operon (Tillett *et al*., 2000), and the fact that the addition of MC to *Microcystis* cultures has been shown to trigger MC production (Schatz *et al*., 2007) and differential expression of cell wall receptor proteins (Kehr *et al*., 2006; Zilliges *et al*., 2008), suggests that these molecules may also play a role in cell signalling.

### Saxitoxins

The carbamate alkaloid neurotoxin, saxitoxin (STX), is the parent compound of at least 57 other naturally occurring analogues (Wiese *et al*., 2010) that are collectively referred to as paralytic shellfish poisoning toxins (PSPs or PSTs) due to their ability to block sodium channels leading to paralysis (Catterall, 1980) and their proclivity to accumulate in shellfish tissue (Negri and Jones, 1995). PSPs in marine environments are produced by the dinoflagellates *Alexandrium*,* Gymnodinium* and *Pyrodinium*, whereas in freshwater environments, potential cyanobacterial producers include *Anabaena*,* Aphanizomenon*,* Cylindrospermopsis*,* Lyngbya*,* Oscillatoria*/*Planktothrix*,* Raphidiopsis* and *Scytonema* (Neilan *et al*., 2013) (Table 1). In recent years, there has been an increase in PSP monitoring efforts in freshwater systems and these efforts have revealed that PSPs may be more widespread than previously believed. For example, the 2007 USEPA National Lakes Assessment identified STXs in 7.7% of inland lakes surveyed (Loftin *et al*., 2016) and Fetscher *et al*. (2015) identified STXs attributable to benthic cyanobacteria in 7% of wadeable streams surveyed in California, USA. A recent study of benthic cyanobacteria in Brazilian rivers and reservoirs identified STX genes and toxin in strains of *Phormidium*,* Cylindrospermum* and *Geitlerinema* for the first time (Borges *et al*., 2015). In a study of Finnish lakes and brackish coastal waters, the genes involved in STX biosynthesis were detected on average in 31% of samples collected (Savela *et al*., 2015).

**Table 1:** Cyanobacterial genera with strains known to be able to produce cyanotoxins

Class	APHA	CYL	DOL	FISC	GLO	LYNG	MIC	NOD	NOST	OSC	PHOR	PLA	RAPH	SCYT
Anatoxin‐a	X	X	X							X	X	X	X	
Cylindrospermopsin	X	X	X										X	
Microcystin			X	X	X		X	X^a^	X	X		X		
Saxitoxin	X	X	X			X						X	X	X

a Produces nodularin – a pentapeptide variant of microcystin.

APHA, *Aphanizomenon*; CYL, *Cylindrospermopsis*; DOL, *Dolichospermum* (formerly *Anabaena*); FISC, *Fischerella*; GLO, *Gloeotrichia*; LYNG, *Lyngbya*; MIC, *Microcystis*; NOD, *Nodularia*; NOST, *Nostoc*; OSC, *Oscillatoria*; PHOR, *Phormidium*; PLA, *Planktothrix*; RAPH, *Raphidiopsis; SCYT*, Scytonema.

The gene cluster (*sxt*) involved in the biosynthesis of STX and its analogues has been sequenced from isolates of *Aphanizomenon*,* Anabaena*,* Cylindrospermopsis*,* Lyngbya* and *Raphidiopsis*. The operons range in size from 26 to 33 genes (spanning ~26–36 kb) with each strain possessing its own arrangement and the specific toxin profile of each strain being determined by the presence or absence of specific genes within the cluster (Neilan *et al*., 2013). It has been hypothesized that the *sxt* gene cluster may have originated in an ancient α‐proteobacterium and has subsequently spread via horizontal gene transfer to diverse lineages of phytoplankton (Kellmann *et al*., 2008). The relative toxicity of STX analogues can be roughly ordered from highest to lowest as: STX> neo‐STX > gonyautoxins (GTX1‐4) > decarbamoylated STXs > di‐sulfated STXs (C1‐C4) (Usleber *et al*., 1997).

In *C. raciborskii*, conductivity has been shown to exhibit a strong positive relationship with PSP production following exposure to high Na^+^ (Pomati *et al*., 2004) or Mg^2+^ concentrations (Kellmann and Neilan, 2007). Gene expression analyses have also shown that expression of *sxtA*, which encodes the first step of STX biosynthesis, and the transporters *sxtF* and *sxtM* are all upregulated in response to Na^+^ stress (Ongley *et al*., 2016). The postulated reason for increased STX production across an ionic gradient is that STX may help the cells regain homoeostasis under salt stress by altering cell permeability such that salt uptake is inhibited (Pomati *et al*., 2003; Brentano *et al*., 2016). Studies on both *Cylindrospermopsis* and *Raphidiopsis* indicate that STX is actively exported out of the cell in response to elevated cation concentrations (Soto‐Liebe *et al*., 2012; Ongley *et al*., 2016).

Nitrogen depletion has been shown to increase STX export in *Aphanizomenon gracile* (Casero *et al*., 2014), whereas temperature seems to have little influence on STX production and export apart from its role in cell growth (Casero *et al*., 2014). Elevated pH above 9 was found to induce significantly higher levels of STX export from cultures of *Anabaena circinalis* and *C. raciborskii* than even elevated conductivity (Pomati *et al*., 2004; Ongley *et al*., 2016), suggesting that alkaline stress may be of central importance in the physiology of STX‐producing strains. Considering that cyanobacterial blooms often bring about significant increases in pH and more alkaline conditions in affected water bodies, STX production may provide a competitive advantage to strains during periods of elevated photosynthetic activity.

## Management implications

It is well established that in most cases, nutrient (both N and P) reductions are required to control cyanobacterial blooms (Paerl, 2014). However, depending on the severity of nutrient over‐enrichment and the size of the water body impacted, these approaches may take years or decades before significant improvements to water quality are achieved (Paerl, 2014). Based on our understanding of the environmental factors promoting toxin production and/or toxigenic strain dominance, there may be other management strategies that can be employed in the meantime – in addition to nutrient reductions – that will favour non‐toxic strain dominance in place of toxin‐producing variants.

Microcystins are preferentially produced under high light intensities, therefore smaller lakes/reservoirs may benefit from the addition of light absorbing dyes during the summer months when cyanobacteria are most proliferative. In riverine systems, riparian buffer and shade along the shoreline should reduce light intensity through the water column, which may also help to reduce MC production. Reduced light intensity should also help to lower water temperature, which may further enhance the competitive advantage of non‐toxic strains. On the contrary, if anatoxin‐a or CYN are problematic, warmer temperatures and higher light intensity may help to alleviate toxin production. In dammed rivers where cold water refugia are not critical for fish habitat, a larger percentage of warmer surface water could be released downstream from dam spillways if benthic cyanobacteria are the suspected toxin producers. Lastly, brackish systems impaired by STXs may benefit from periodic pulses of freshwater to lower ionic potential or the addition of dilute acids to lower pH during periods of high cyanobacterial productivity. While these suggestions are purely experimental at this time, they are provided to illustrate how an improved understanding of cyanobacterial ecophysiology and toxin production may one day lead to novel management strategies for mitigating cyanobacterial toxicity in surface waters.

## Conclusions

**Figure 1:**
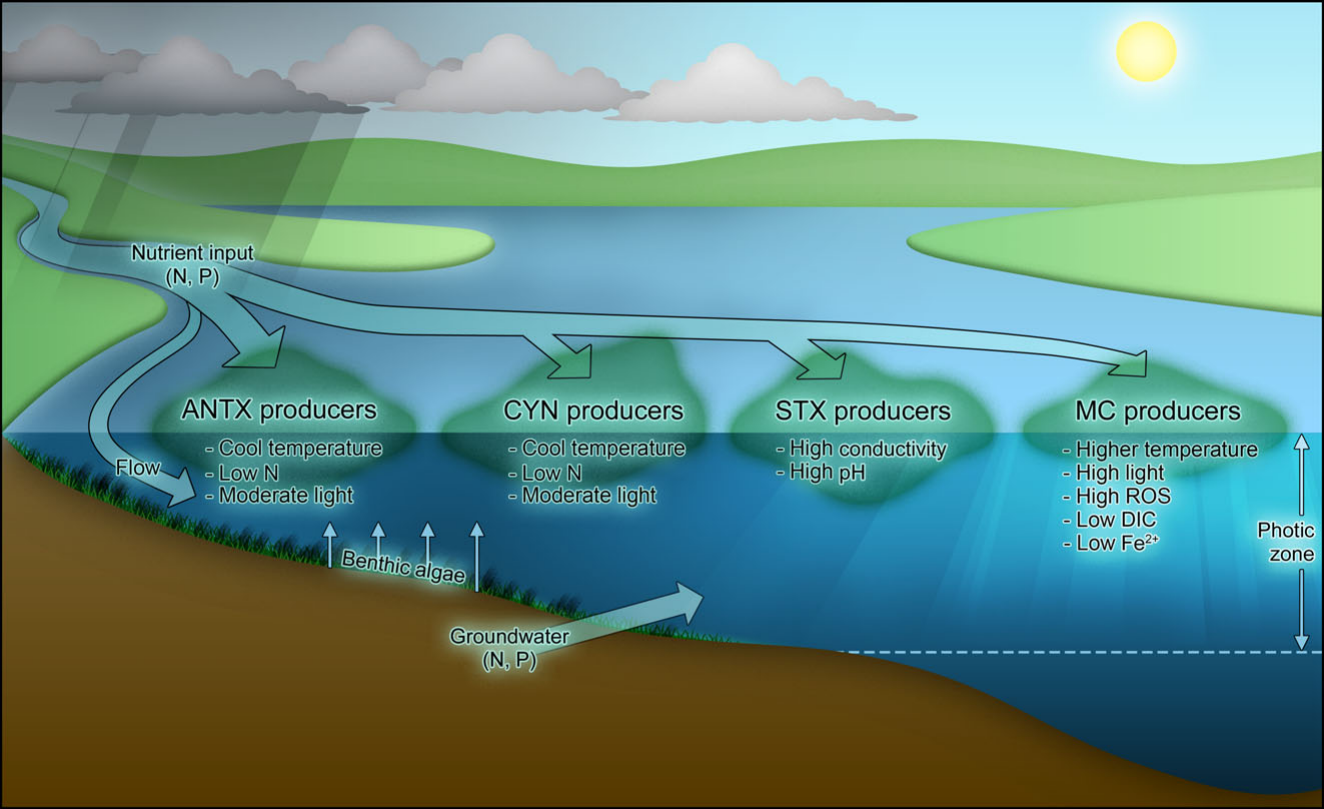
Putative environmental drivers of cyanotoxin production that are presently recognized and the interplay of environmental stimuli on growth of toxigenic cyanobacteria.

Studies investigating the ecological and physiological roles of cyanotoxins indicate that there are likely to be multiple functions performed by each of these molecules. Figure 1 attempts to convey the most commonly observed drivers for production of each cyanotoxin class under natural settings. Much of our understanding of cyanotoxins comes from culture studies using so‐called toxic and non‐toxic strains. However, based on the several dozen cyanobacterial genomes that have been sequenced to date, it is now apparent that there are significant genomic differences between even seemingly closely related strains. In *Microcystis aeruginosa*, the core genome – defined as those genes found in all strains of *M. aeruginosa* – is estimated to only be ~2400 genes, or ~47% of the genome (Humbert *et al*., 2013). As such, any studies attempting to compare different strains should be cognizant that there may be considerable differences in their overall genetic makeup, which will influence their ability to adapt to varied environmental conditions (Harke *et al*., 2016a). At present, *M. aeruginosa* is the only HAB‐forming cyanobacterium for which isogenic mutants (e.g. Δ*mcyB*‐) have been successfully created, thereby allowing direct assessments of the physiological function of MC in this strain. As new gene editing tools, such as various CRISPR‐Cas systems evolve (Selle and Barrangou, 2015; Yao *et al*., 2016), genetic manipulation of other genera of cyanobacteria should become accessible.

In the meantime, much can be inferred about the putative functions of cyanotoxins by studying cyanobacterial blooms *in situ* using a variety of ‐omics techniques. It is now possible to extract nearly complete cyanobacterial genomes from shotgun sequencing data sets using differential binning approaches (Albertsen *et al*., 2013; Brown *et al*., 2016; Otten *et al*., 2016). With reference genomes in hand that are specific to the system being investigated, metatranscriptomics can be used to massively survey the system and the resulting gene transcripts corresponding to CyanoHAB taxa can be mapped back to their cognate hosts. This methodology performed over a time‐series will provide unparalleled insights into cyanobacterial physiology and toxin production that cannot be obtained using cultured isolates. These methods are starting to gain traction (e.g. Penn *et al*., 2014; Harke *et al*., 2016b) and in the coming years we anticipate an explosion of metagenomic, metatranscriptomic and proteomic data which will usher in a new understanding of the drivers of bloom initiation, collapse and toxicity.
